# Supplementary material for: MetaRibo-Seq measures translation in microbiomes
Source: Nat Commun. 2020 Jun 29;11:3268. doi: 10.1038/s41467-020-17081-z (PMC7324362; doi:10.1038/s41467-020-17081-z)
Supplement: Supplementary file 10 — Supplementary Data 7 [file 41467_2020_17081_MOESM10_ESM.zip › File2/Confidence_High_Taxonomy/487_out.krona.html]

Javascript must be enabled to view this page.

members
magnitude
magnitudeUnassigned
count
unassigned
taxon
rank

487\_out

3

superkingdom
3
2

1
201174
phylum

class
1
1760

1
85006
order

family
1
1268

genus
32207
1

species

SRS011126\_contig\_number\_10775
172042
1

976
1
phylum

class
1
200643

order
171549
1

171552
1
family

838
1
genus

species

SRS143135\_contig\_number\_384
1
228604

phylum
1
1239

909932
1
class

order
909929
1

1843491
1
family

1
970
genus

1
712538
species

SRS893322\_contig\_number\_15832
